# Deciphering the role of CCL4-CCR5 in coronary artery disease pathogenesis: insights from Mendelian randomization, bulk RNA sequencing, single-cell RNA, and clinical validation

**DOI:** 10.7150/ijms.99518

**Published:** 2024-10-14

**Authors:** ZiAn Feng, Hui Li, Nan Chen, Kai Xu, BuChun Zhang

**Affiliations:** 1 Department of Cardiology, The First Affiliated Hospital of USTC, Division of Life Sciences and Medicine, University of Science and Technology of China, Anhui Hefei, 230001, China.; 2 Graduate School, Wannan Medical College, Anhui Wuhu, 241002, China.; ZiAn Feng and Hui Li contribute equally to this work.

**Keywords:** CCL4, Mendelian randomization, Inflammatory cytokines, Coronary artery disease, Single-cell RNA sequencing

## Abstract

**Background:** Alterations in circulating CCL4 levels have been implicated in coronary artery disease (CAD), but the causal relationship and underlying mechanisms remain unclear.

**Objective:** This study aims to analyse the role of CCL4 and its receptor (CCR5) in CAD using Mendelian randomisation (MR) analysis, bulk RNA and single cell RNA sequencing (scRNA-seq).

**Methods:** The MR analysis was used to determine the causal relationship between 91 circulating inflammatory proteins and CAD. Bulk RNA sequencing data was used to demonstrate the expression profile of CCL4/CCR5. The localisation of CCL4/CCR5 was determined using scRNA-seq data. Functional enrichment analyses were used to infer the potential role of CCL4 in CAD. Additional clinical samples were utilized to validate the results of MR.

**Results:** We identified six circulating inflammatory proteins associated with CAD. Of these, CCL4 was identified as a key inflammatory cytokine associated with CAD risk for MR analysis.The bulk RNA sequencing data from the Gene Expression Omnibus (GEO) datasets showed that CCR4 receptor(CCR5) expression was significantly higher in human atherosclerotic plaques compared to controls. Notably, scRNA-seq analysis revealed CCL4 was highly expressed in T cells, monocytes and macrophages. Clinical specimens confirmed high levels of serum CCL4 expression in CAD patients by ELISA.Functional enrichment analysis revealed that CCL4 was primarily enriched in the cytokines and cytokine receptors, viral proteins with cytokines and cytokine receptors, and chemokine signaling pathways.

**Conclusion:** Our study presented a genetic insight into the pathogenetic role of CCL4-CCR5 in CAD, which may provide new insights for further mechanistic and clinical investigations of inflammatory cytokine-mediated CAD.

## Introduction

Coronary artery disease (CAD) is characterised as a form of arterial inflammation. Clinical epidemiological research has shown that high levels of pro-inflammatory cytokines are often associated with CAD^1^. In particular, increased circulating levels of the chemokine C-C motif ligand 4 (CCL4) have been observed in individuals with CAD^2^. However, despite these associations found in observational studies, a causal relationship with CAD cannot be confirmed due to potential confounding factors.

Mendelian randomisation (MR) is a powerful genetic technique used to identify causal risk factors for CAD. It is based on the concept that genetic variants are randomly distributed and independent of other disease risk factors^3^. Several studies have validated the MR approach in delineating causal associations between serum biomarkers and CAD severity^4^. In addition, this study will integrate single-cell RNA sequencing (scRNA-seq) and bulk RNA sequencing(bulk RNA-seq) data to fully elucidate the potential mechanisms of CCL4 in CAD development. scRNA-seq technology provides the capability to analyze changes in specific cell types during the progression of disease^5^. Meanwhile, bulk RNA-seq can provide overall gene expression information for further validation and refinement of single cell transcription^6^.

In our research, we first performed an MR analysis using aggregated GWAS data for inflammatory cytokines in relation to CAD to assess the causal impacts of CCL4 on the disease. We then explored both bulk RNA-seq and scRNA-seq data to unravel the potential role of CCL4 and its receptor(CCR5) in the development of atherosclerosis and CAD. For validation, clinical blood samples from CAD patients were analysed. Through these comprehensive genetic and immunological analyses, we aim to elucidate the role of circulating CCL4 as a causal biomarker for CAD.

## Methods

### Study design

**Figure 1:**
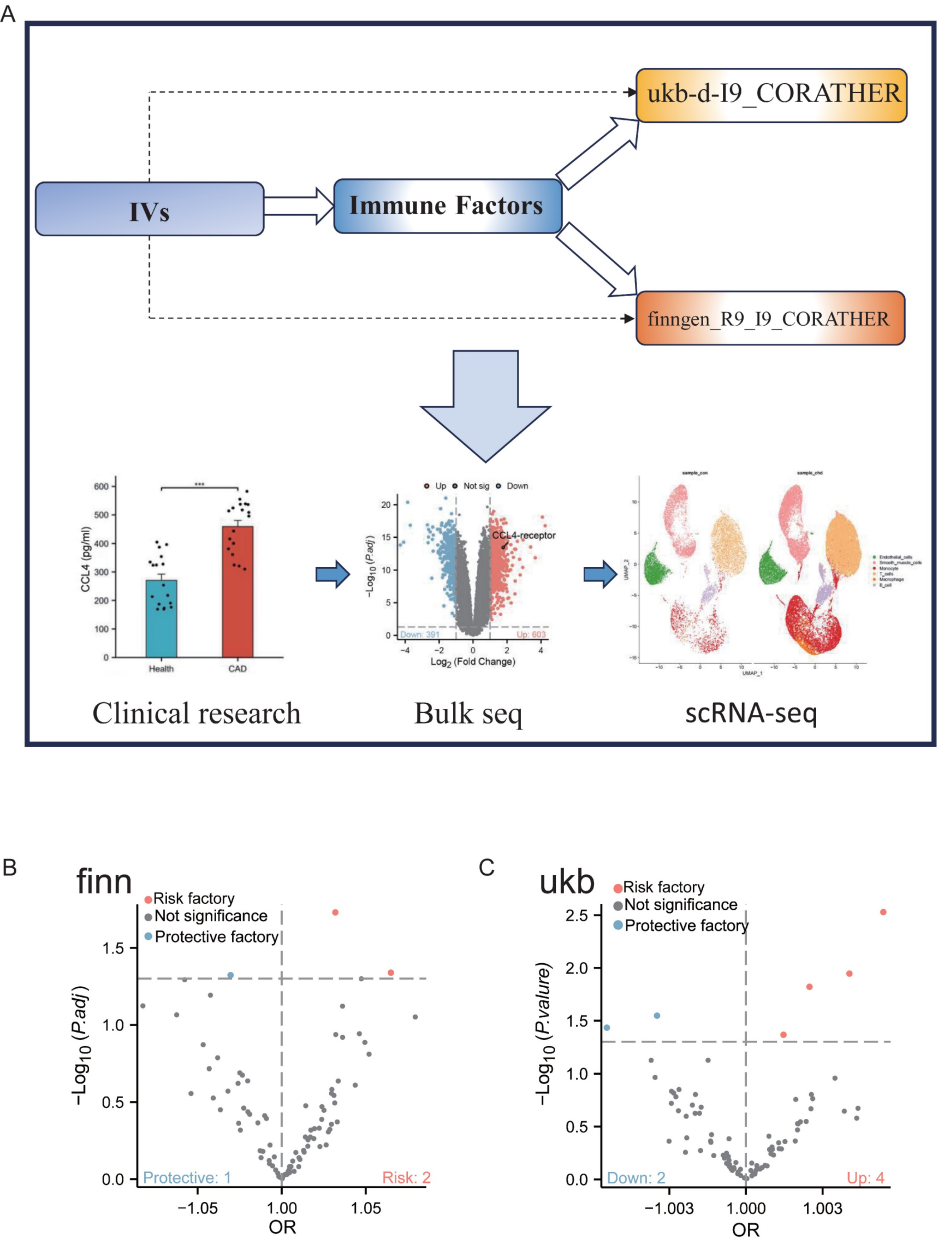
Flowchart of study design. A. Two-sample Mendelian randomisation (MR) was used to infer causality between CCL4 exposure and CAD outcomes, transcriptomics data from RNA-seq and scRNA-seq, and clinical serum samples from patients with atherosclerosis were analysed. B. Genome-wide association study (GWAS) data for inflammatory cytokines were obtained from the FinnGen consortium. C. Genome-wide association study (GWAS) data for inflammatory cytokines were obtained from the UK Biobank. CCL4, C-C chemokine ligand 4; CAD, coronary artery disease; IVs, instrumental variables; RNA-seq, bulk RNA sequencing; scRNA-seq, single cell RNA sequencing; GWAS, genome-wide association studies.

Figure 1 depicts the schematic overview of our research approach. The objective of this study was to investigate the causal relationships between circulating CCL4 proteins and CAD through MR analysis. In addition, we employed serum samples and single-cell transcriptomic data from clinical specimens to confirm the findings derived from our MR analysis.

### GWAS data sources

In the present study, summary level statistics for both exposure (inflammatory cytokines) and outcome (CAD) were derived from European ancestry, which may reduce bias due to race-related confounders. GWAS summary statistics for levels of 91 circulating cytokines were derived from the largest and most recent genome-wide protein quantitative trait locus (pQTL) study available, which includes 14,824 individuals of European ancestry from 11 independent cohort studies^7^. Information on these 91 plasma inflammatory proteins is available in the EBI GWAS catalogue (accessions GCST90274758 through GCST90274848). The summary statistics for CAD in the GWAS were obtained from the FinnGen consortium(https:// www.finngen.fi/en) and the UK Biobank(https://www. ukbiobank. ac.uk/), with a total of 37,697 cases of CAD and 534,700 controls^8^.

### Instrument selection

We identified single nucleotide polymorphisms (SNPs) significantly associated with CAD (*P*<1e-05) but not with cytokine levels (P>0.05) to serve as candidate instrumental variables (IVs) for MR. These candidate IVs were selected based on three critical MR assumptions: (1) the IVs are significantly associated with CCL4 (*P*<1e-05), (2) the IVs are not linked to any confounders, and (3) the IVs do not correlate with CAD outcomes (*P*>0.05). Additionally, to ensure the independence of these IVs, they were further refined by pruning linkage disequilibrium (LD) coefficients (r^2^ <0.01).

### Mendelian randomization analysis

We identified SNPs suitable as MR IVs from the GWAS data for Covid and Sevcovid based on genome-wide significance criteria (*P<*5×10^^^-8) and stringent linkage disequilibrium standards (LD r^^^2 = 0.001 and kb = 10000). These SNPs were selected for their strong association with the exposure without correlation to the outcome and were independent of known confounders. The strength of these IVs was verified by F-statistics>10. To adhere to MR's independence and exclusion restriction assumptions, SNPs linked to age, smoking, alcohol use, body mass index, blood glucose, lipid levels, and other possible confounders affecting AMI were excluded using the PhenoScanner tool^9^. This study used the FinnGen and UK Biobank datasets for analysis. All of the analysis methods, conditions and filter thresholds were the same, and we adopted this approach to increase the reliability of our findings. We assessed IV heterogeneity using Cochran's Q-statistic and checked for horizontal pleiotropy with the Egger intercept test (*P* < 0.05). All procedures were performed in R software (version 4.2.2).

### Bulk RNA data analysis

We analyzed two RNA-seq datasets (GSE100927 and GSE28829^10^,^11^) from the Gene Expression Omnibus (GEO) to evaluate the expression levels of the CCL4 receptor (CCR5) in human atherosclerotic plaques. The selection criteria for the bulk RNA-seq data included datasets with both cases and controls, each consisting of at least 10 samples. Only genes with an adjusted *P* value < 0.05 and |logFC(fold change)| ≥1 were identified as differentially expressed genes(DEGs). The ggplot2 package was used to generate volcano plots for the visualisation of the DEGs.

### Human serum samples collection

Patients with CAD in this study were recruited from our prospective registry study of patients undergoing percutaneous coronary intervention (PCI) between October 2022 and December 2024(registered on the Chinese Clinical Trial Registry [ChiCTR]2200064634). Peripheral whole blood was collected from 19 patients diagnosed with CAD via coronary angiography and 27 individuals without CAD. Patient's characteristics, laboratory tests including biochemical parameters and traditional cardiovascular risk factors are summarized in Table 1. This research work was approved by the medical research ethics committee of our institution (Approval no: 2024KY093). The serum samples were collected from the participants in the fasting state.

### Serum CCL4 proteins measurement

The amount of human CCL4 protein was determined using an enzyme-linked immunosorbent assay (ELISA) kit (Ruixin Biological Technology Co. Ltd, Quanzhou, China). All serum samples were diluted 1:10 with ELISA buffer. The assays were performed in triplicate according to the manufacturer's instructions. Serum CCL4 concentrations were calculated using standard curves generated from CCL4 standards supplied with the kit.

### Single-cell RNA data analysis

Single-cell RNA-seq data covering human atherosclerotic plaques and adjacent normal tissues were obtained from Alsaigh T *et al.*^12^ and are available in the GEO under accession number GSE159677. Quality control measures were initially applied to both samples and cells, using criteria such as cell count per sample, number of features per cell and mitochondrial gene content per cell. Samples with fewer than 1000 cells were excluded to ensure sufficient cell numbers for subsequent analyses. For individual cells, we assessed the number of features and the proportion of mitochondrial genes. Only cells that met the quality standards of more than 200 features and less than 20% mitochondrial genes, according to standard quality control parameters, were retained. After quality control, we integrated the high quality samples and normalised the raw RNA counts using the SCTransform function from the Seurat R package (version 4.0.5). Log normalised RNA counts were then used for further analysis. Cells were then clustered by principal component analysis (PCA) using the RunPCA function. To identify the major cell types, PCA was performed on 2000 highly variable genes using the top 50 principal components. The cell clusters were annotated based on the marker genes reported in the original study. The clusters were visualised using UMAP plots. In addition, data on cytokine-related pathways were obtained from the Molecular Signatures Database (MSigDB) 13.

### Functional and pathway enrichment analysis

To analyze the functions of differentially expressed genes (DEGs) between the high-CCL4 and low-CCL4 groups, we employed Gene Ontology (GO) enrichment and Kyoto Encyclopedia of Genes and Genomes (KEGG) pathway analysis. In our statistical analysis, a pathway was considered to be statistically significant when *P* < 0.05.

## Results

### Identification of the most prominent pro-inflammatory cytokines associated with coronary artery disease

**Figure 2:**
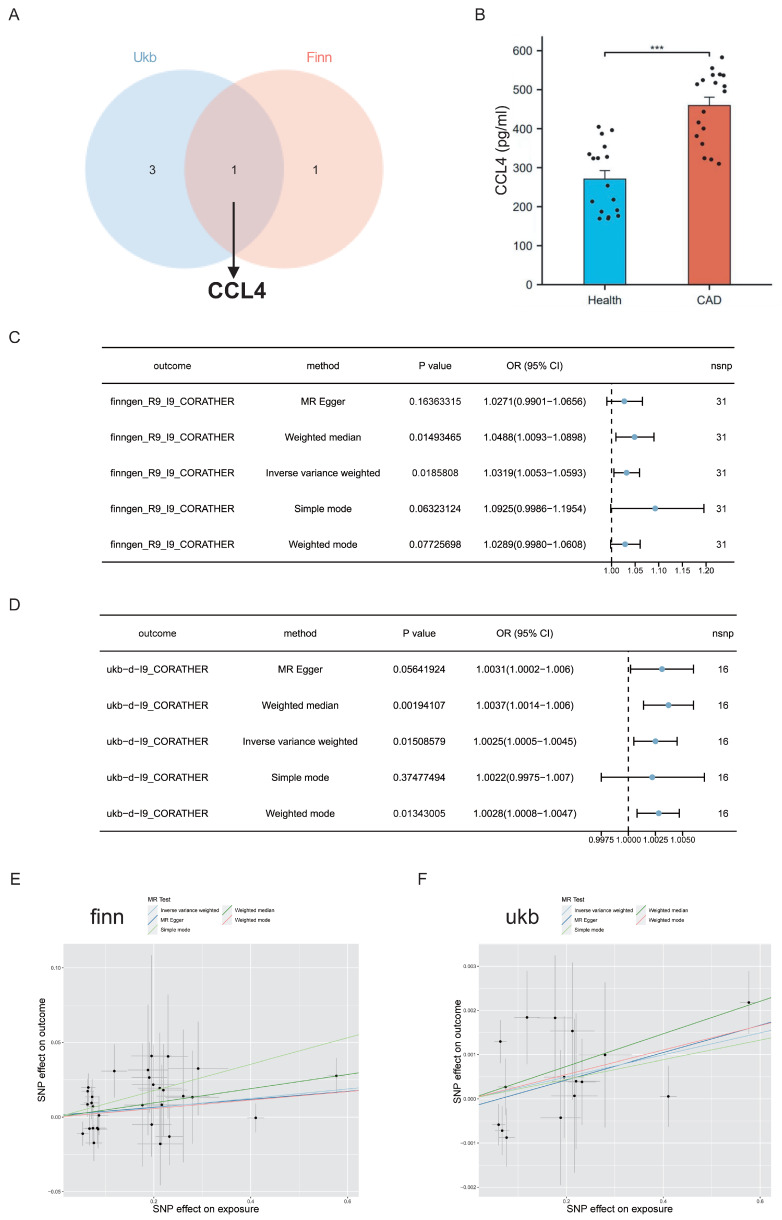
Evaluation of the causal effects of CCL4 levels on CAD. A. Venn diagram showing the CCL4 genes shared by FinnGen consortium and UK Biobank. B.Expression of serum CCL4 levels in CAD patients using ELISA. C. Forest plot of two-sample MR analyses of CCL4 and the risk of CAD in the FinnGen database. D. Forest plot of two-sample MR analyses of CCL4 and the risk of CAD in the UK Biobank database. E. Scatter plot of causal effect for CCL4 on CAD in the FinnGen database. F. Scatter plot of causal effect for CCL4 on CAD in the UK Biobank database. CCL4, C-C chemokine ligand 4; CAD, coronary artery disease.

To identify the most critical pro-inflammatory cytokines associated with CAD, we constructed a Venn diagram based on overlapping genes. In this study, six circulating inflammatory proteins served as IVs for CAD, four from the UK Biobank and two from the FinnGen consortium. Notably, CCL4, which is common to both databases, was identified as a key cytokine (Figure 2A). As a result, CCL4 was recognised as a key inflammatory cytokine and selected for further analysis.

### Identification of elevated levels of CCL4 in clinical blood samples from patients with coronary artery disease

Serum CCL4 levels were measured by ELISA in a cohort of 19 CAD patients and 27 non-CAD subjects. The results showed significantly elevated levels of CCL4 in the CAD group (Figure 2B), highlighting its important role in the pathogenesis of the disease.

### Genome-wide mendelian randomization identified the causal effects of CCL4 on coronary artery disease

MR analysis using the inverse variance weighted (IVW) method showed a significant association between elevated CCL4 levels and an increased risk of CAD in both the FinnGen (OR=1.0319, 95% CI 1.0053-1.0593; *P* =0.0185808) and UK Biobank (OR =1.0025, 95% CI 1.0005-1.0045; *P*=0.0.01508579) databases. The MR-weighted median approach further confirmed this association in both databases. In addition, results from other MR techniques pointed in the same direction, suggesting a consistent trend, although these were not statistically significant (Figure 2C,D). The scatter plots illustrating the causal effects of CCL4 on CAD suggested a trend towards a positive correlation. This trend is consistent with the forest plots of the causal effect estimates from the MR analysis (Figure 2E, F).

### Increased CCR5 expression in human atherosclerotic lesions

**Figure 3:**
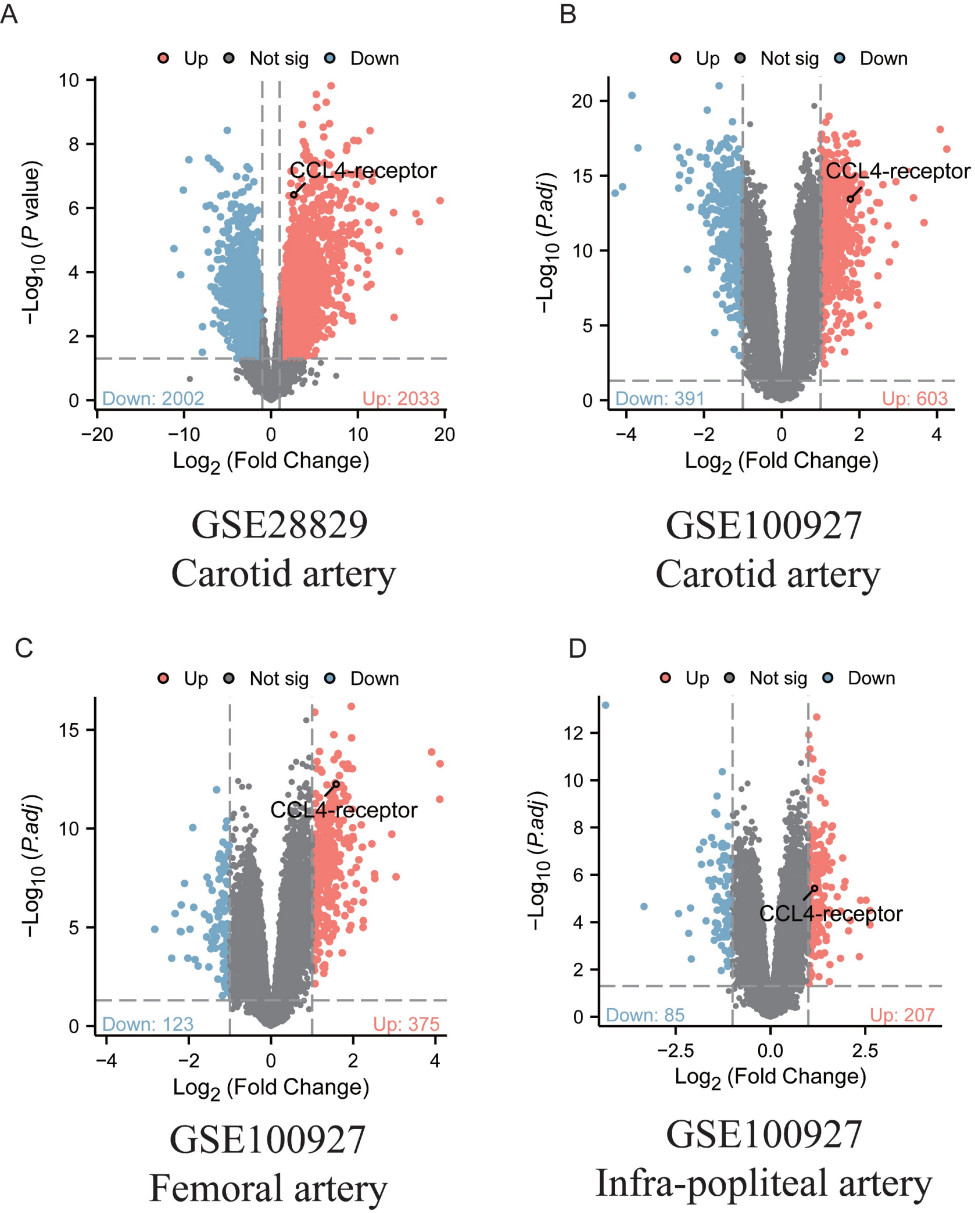
Analyses of CCL4-receptor expression from human atherosclerotic plaques. A.Volcano map of differentially expressed genes in GSE28829. B-D:Volcano map of differentially expressed genes in GSE100927. Red dot: upregulated gene, blue dot: downregulated gene.

Ligands and receptors represent a class of molecules that are accessible for the development of therapeutic agents. To assess changes in CCL4 receptor (CCR5) expression within human atherosclerotic plaques, two bulk RNA-seq datasets (GSE28829 and GSE100927) were analysed. The analysis revealed that CCL4 receptor expression was significantly upregulated in atherosclerotic plaque samples compared to control samples (Figure 3). The increased expression of CCL4 receptor further support MR findings.

### Analyses of single-cell RNA-seq data from human atherosclerotic plaques

**Figure 4:**
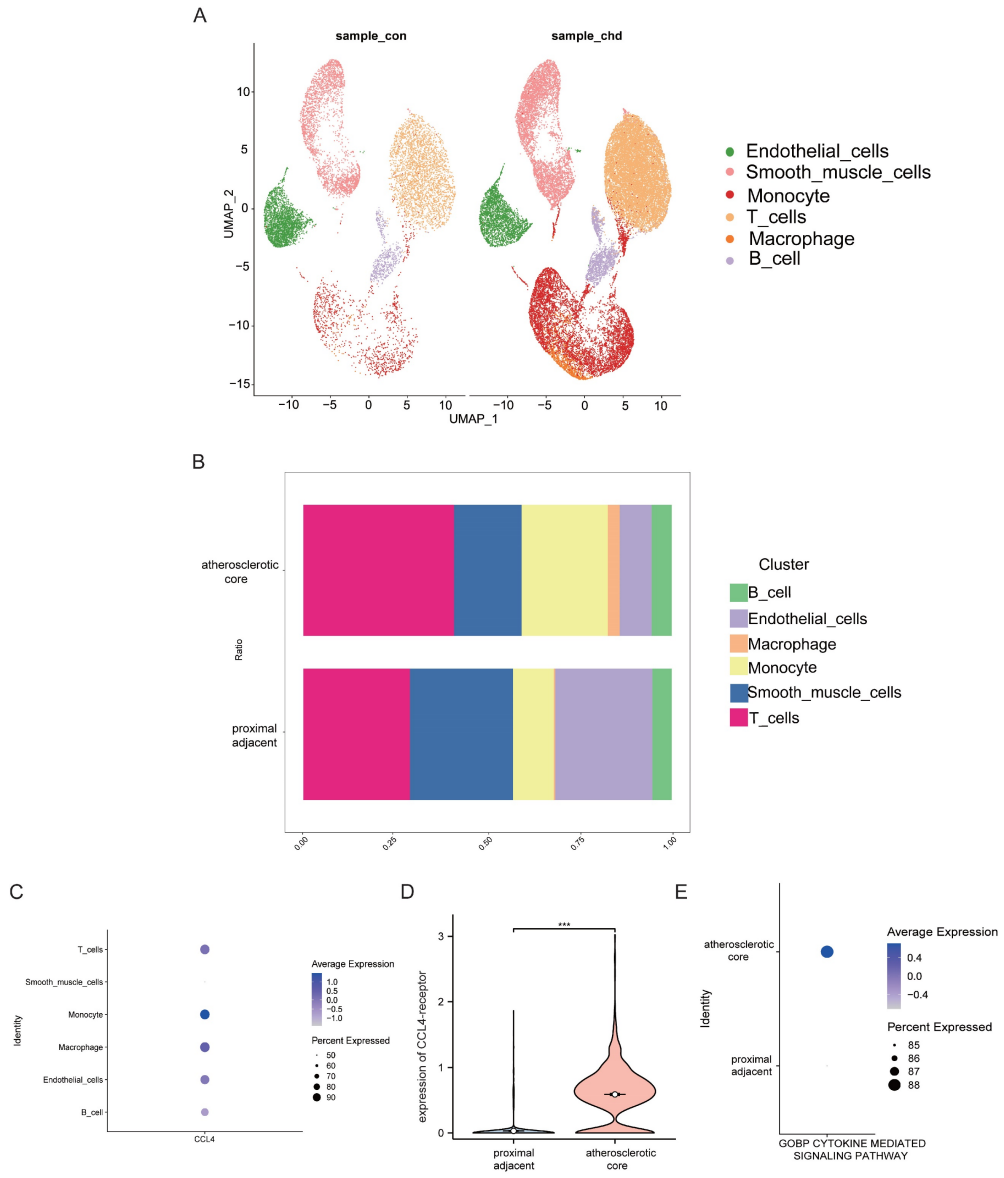
Analyses of single-cell RNA-seq data from human atherosclerotic plaques. UMAP plot showing six major cell types. B. Barplot showing the contribution of cell types to two groups. C. Bubble plot showing the expression level of CCL4 across six cell subtypes. D. Violin plot showing the expression of CCL4-receptor between two groups. E. Bubble plot showing the cytokine-mediated signaling pathways between two groups. CCL4, C-C chemokine ligand 4.

To investigate CCL4 expression at the cellular level in atherosclerotic plaques, we performed an analysis using scRNA-seq data. This analysis identified seven distinct cell clusters within human atherosclerotic plaque tissue, including endothelial cells, smooth muscle cells, monocytes, T cells, macrophages and B cells (Figure 4A). We then assessed CCL4 expression in different immune cell subtypes and found CCL4 highly expressed in T cells, monocytes and macrophages within atherosclerotic plaques compared to controls (Figure 4B,C). In addition, a significant increase in CCL4 receptor (CCR5) gene expression was observed in macrophages from atherosclerotic plaques(Figure 4D). In particular, cytokine-mediated signalling pathways were more active in atherosclerotic plaques than in surrounding vascular cells (Figure 4E). These results highlight the important role of CCL4-CCR5 in immune cells that may influence atherosclerotic plaque progression.

### Functional enrichment analysis of CCL4 gene in atherosclerotic plaques

**Figure 5:**
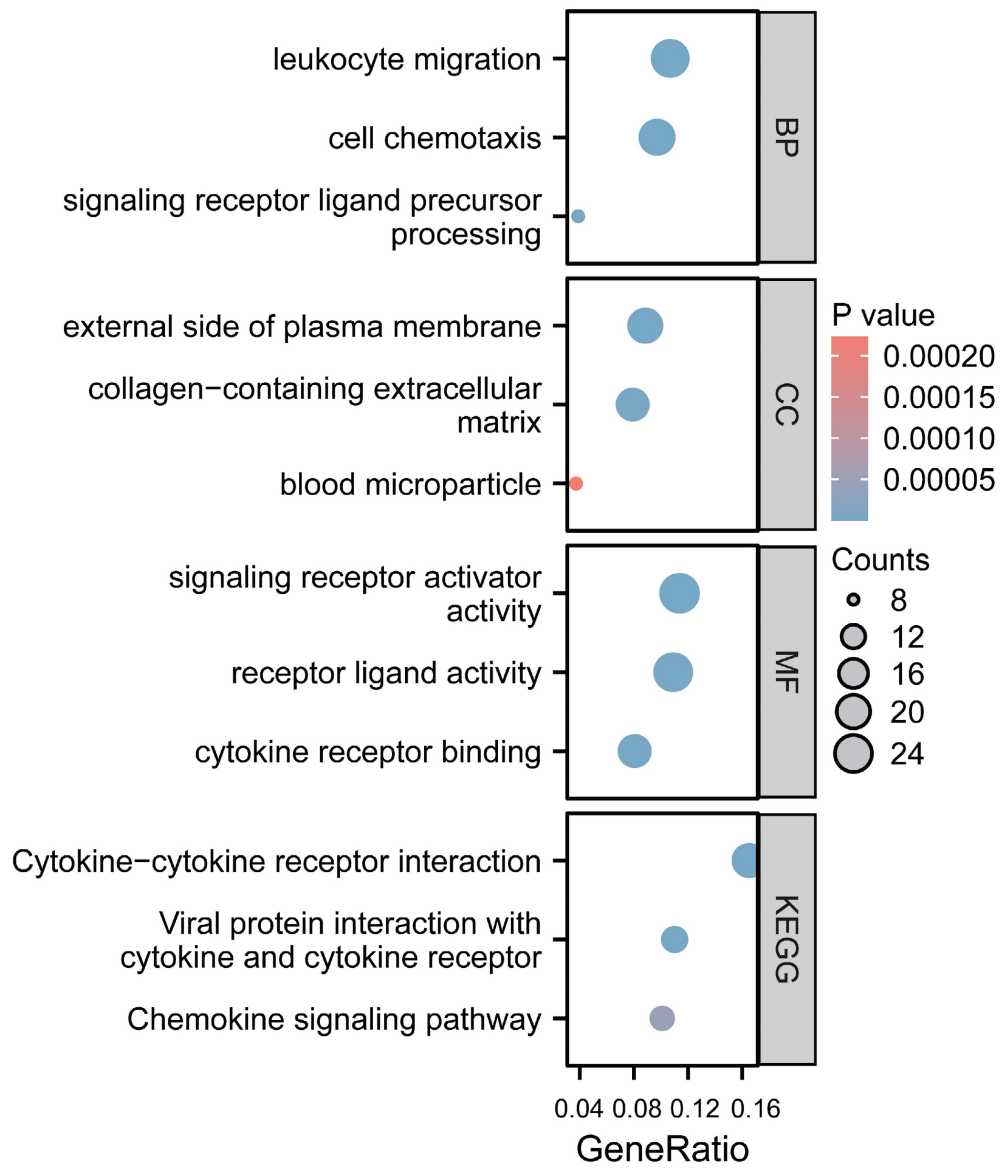
Functional analysis of CCL4 gene in atherosclerotic plaques. CCL4, C-C chemokine ligand 4.

We employed GO function and KEGG pathway enrichment analyses to elucidate the biological functions of the CCL4 gene in atherosclerotic plaques. The top three significantly enriched GO categories-biological process (BP), molecular function (MF), and cellular component (CC) are highlighted. The BP category showed that CCL4 is involved in leukocyte migration and cell chemotaxis. Notably, the most significantly enriched MF pathways included signaling receptor activator activity, receptor ligand activity, and cytokine receptor binding. KEGG analysis identified the top pathways as cytokine-cytokine receptor interaction, viral protein interaction with cytokine and cytokine receptor, and the chemokine signaling pathway (Figure 5).

## Discussion

In this study, six circulating inflammatory proteins-four from the UK Biobank and two from the FinnGen consortium were used as IVs for CAD. Using a Venn diagram to analyse the overlap of key proteins, CCL4 emerged as a key cytokine and was further analysed. Our MR results showed an association between higher CCL4 levels and increased CAD risk. ELISA tests confirmed that serum levels of CCL4 were significantly elevated in patients with CAD compared to controls. Our analysis of single-cell RNA sequencing data showed that CCL4 was upregulated in T cells, monocytes and macrophages derived from atherosclerotic plaques. Analysis of bulk RNA sequencing data from the GEO datasets showed that the expression of CCR4 receptor (CCR5) was significantly increased in human atherosclerotic plaques compared to control samples.

We analysed genetic, transcriptomic and clinical data to identify the role of CCL4 in the development of CAD. By performing MR analysis, we established a causal relationship between CCL4 and CAD. By integrating scRNA-seq and bulk RNA-seq data, we mainly describe the role of CCL4 in the process of CAD from two aspects. First, at the single cell level, we found that immune cells in atherosclerotic plaques produce CCL4. Second, at the bulk RNA-seq level, we found that CCR5 is present in human atherosclerotic plaques. Clinical data from CAD patients also suggest that CCL4 may be associated with a higher risk of CAD.

Atherosclerosis is characterised by the local accumulation of apolipoprotein B-containing lipoproteins, immune cells, vessel wall cells and extracellular matrix beneath the endothelium. These lipoproteins adopt characteristics similar to damage-associated molecular patterns, initially triggering an innate immune response led by monocyte-macrophages, followed by an adaptive immune response^14^. These inflammatory processes often become chronic, leading to non-resolving conditions that can cause arterial damage and thrombosis-induced organ infarction^15^. Regulation of the innate immune response occurs at several stages, including haematopoiesis, monocyte transformation and macrophage activation, whereas the adaptive immune response is largely controlled by mechanisms that modulate the balance between regulatory and effector T cells^16^. In this study, our scRNA-seq data revealed a significant upregulation of the CCL4 gene in T cells, monocytes and macrophages within atherosclerotic plaques. The progression and destabilisation of atherosclerotic plaques is driven by the activated macrophages and T cells and the cytokines that mediate their interactions. Consistent with our work, previous studies have also shown that CCL4 is predominantly expressed in monocytes/macrophages and T cells from human carotid atherosclerotic plaques^17^. Several studies have shown that monocytes, macrophages and T cells release more CCL4 under inflammatory microenvironment conditions to promote atherosclerosis formation and plaque progression, further explaining that CCL4 secreted by immune cells maintains chronic inflammatory responses within atherosclerotic plaques^18^,^19^.

CCL4 is a chemokine that binds to the receptor encoded by CXCR5 and plays an important role in the inhibition of vascular endothelial cell growth, regulation of platelet aggregation, modulation of inflammatory responses, immunomodulation and angiogenesis 20. CCL4 has been extensively studied in the context of atherosclerosis and has been shown to be highly expressed in several cell types important in CAD, such as T cells, macrophages and endothelial cells of atherosclerotic plaques^21^. Experimental data from animal models of atherosclerosis suggest that direct inhibition of CCL4 stabilises atherosclerotic plaques and reduces endothelial cell and macrophage activation^22^. As reported by others^23^, we observed that CCL4 receptors are also expressed in human atherosclerotic plaques. Together with our MR estimates, these data support a causal relationship between higher CCL4 levels and CAD risk.

Dysregulation of the immune response is a key factor in patients with CAD, particularly in those with underlying disease 24. Clinical studies have shown that the severity of CAD correlates with levels of inflammatory cytokines, including chemokines 25,26. However, the causal relationship between inflammatory proteins and CAD remains to be established. In this context, our MR analysis identified a significant causal relationship between CCL4 levels and CAD risk. In addition, our scRNA-seq data showed that CCL4 expression was significantly upregulated in T cells, monocytes and macrophages within atherosclerotic plaques. This finding supports the results of our MR analyses and provides a molecular mechanism for the increased expression of CCL4 and its receptor during atherosclerotic plaque progression.

Given the critical role of inflammatory and immune cells in the aetiology of atherosclerosis and CAD, it is not surprising that CCL4 and CCR5 have been associated with this disease. They are part of the immune response system and are involved in the recruitment of immune cells to sites of inflammation, a key process in the development of CAD^14^. Research has shown that CCL4 is a chemokine that binds to CCR5, and this interaction can lead to the migration of immune cells such as monocytes and T-cells into the arterial wall. This process contributes to the formation of atherosclerotic plaques, which can narrow the arteries and reduce blood flow, leading to CAD^27^. CCR5 is expressed on various cell types, including macrophages and T cells, and its activation by CCL4 can promote inflammation and the release of cytokines that further contribute to plaque formation and instability^28^.

Through functional enrichment analysis, we identified cytokine-cytokine receptor interaction signaling, viral proteins with cytokines and cytokine receptors, and chemokine signaling pathways involved in CCL4-mediated atherosclerotic plaque formation. The above key pathways have been shown to be important regulators of inflammatory cytokines that influence T cells and macrophages activation in atherosclerosis^29^-^30^.

MR is a powerful statistical technique that uses genetic variants as IVs to estimate the causal effect of risk factors on disease outcomes, reducing the potential for confounding and reversibility bias that can affect traditional observational studies^31^. Here, using the FinnGen and UK Biobank data, we show by MR that high circulating levels of CCL4 are causally associated with an increased risk of CAD. This approach supports our hypothesis that CCL4 may play a direct causal role in the pathogenesis of CAD, particularly through its interaction with CCR5.

Targeting the CCL4-CCR5 interaction for the treatment of CAD could have several implications based on the study's findings: targeting this interaction could potentially modulate the inflammation and immune response associated with the disease. For example, CCR5 antagonists are already used to treat HIV and some cancers and could be repurposed or developed as a treatment for CAD by inhibiting the effects of CCL4. However, it's important to note that the relationship between CCL4, CCR5 and CAD is complex and not fully understood. Further research would be needed to establish a clear link and to determine the efficacy and safety of targeting the CCL4-CCR5 interaction in the treatment of CAD.

### Strengths and limitations

This study has several strengths. Firstly, the predominant strength is the MR design, which estimates the causal effects of CCL4 on CAD without the interference of residual confounding or reverse causality. Secondly, we use multiple statistical methods and data sources to investigate the potential causal relationship and molecular mechanisms between CCL4 and CAD. However, some limitations should be acknowledged. Firstly, no functional experiments have been performed to verify the causal role of CCL4 in CAD. Further studies at the cellular level and in animal models are needed to firmly establish a link between CCL4 and the development of CAD. Secondly, our work on MR analysis was limited to people of European descent. Future research should include diverse populations to validate our findings across ethnic groups. Thirdly, this study did not investigate the effect of blocking the interaction between the CCL4 ligand and its receptor on the immune responses in people with CAD. A goal for future research is to determine the effects of such inhibition on the immune response.

## Conclusions

In summary, we found that higher expression levels of CCL4 were significantly correlated with an increased risk of CAD using MR analysis. Further analysis of the scRNA-seq dataset showed that CCL4 was highly expressed in T cells, monocytes and macrophages within atherosclerotic plaques compared to controls. In addition, CCR5 levels were increased in human atherosclerotic plaques. Our results suggesting that blocking the CCL4-CCR5 interaction may be a promising therapeutic target for CAD.

## Supplementary Material

Supplementary table.

## Figures and Tables

**Table 1:** Baseline characteristics of the CAD patients from the validation cohort

Variables	Control group (n=27)	CAD group (n=19)	*P* value
Age(years), Mean ± SD	60.70 ± 10.30	64.63 ± 8.69	0.18
Male, n (%)	14 (30.4%)	10 (21.7%)	0.96
BMI (kg/m2), Median (IQR)	26 (22.35, 27.85)	25.8 (24.65, 28.4)	0.55
Systolic blood pressure, Mean ± SD	134.41 ± 15.62	143.16 ± 21.72	0.12
Diastolic blood pressure, Mean ± SD	79.37 ± 10.35	78.05 ± 8.55	0.65
Hypertension	15 (32.6%)	14 (30.4%)	0.21
Diabetes	7 (15.2%)	10 (21.7%)	0.07
FBG, mmol/L	4.85 (4.52, 5.40)	6.16 (4.75, 6.83)	0.09
TC, mmol/L	4.21± 0.88	3.97 ± 0.92	0.38
TG, mmol/L	1.26 (0.81, 1.73)	1.25 (1.01, 1.75)	0.71
HDL-C, mmol/L	1.12 ± 0.21	1.00 ± 0.20	0.07
LDL-C, mmol/L	2.58 ± 0.68	2.21 ± 0.68	0.10

CAD: coronary artery disease; BMI: body mass index; FBG: fasting blood glucose; Scr: serum creatinine; TC:total cholesterol; TG: triglyceride; HDL-C: high-density lipoprotein cholesterol; LDL-C: low-density lipoprotein cholesterol.
